# Nuclear β-catenin translocation plays a key role in osteoblast differentiation of giant cell tumor of bone

**DOI:** 10.1038/s41598-022-17728-5

**Published:** 2022-08-04

**Authors:** Atsushi Kimura, Yu Toda, Yoshihiro Matsumoto, Hidetaka Yamamoto, Kenichiro Yahiro, Eijiro Shimada, Masaya Kanahori, Ryunosuke Oyama, Suguru Fukushima, Makoto Nakagawa, Nokitaka Setsu, Makoto Endo, Toshifumi Fujiwara, Tomoya Matsunobu, Yoshinao Oda, Yasuharu Nakashima

**Affiliations:** 1 Department of Orthopedic Surgery, Graduate School of Medical Sciences, Kyushu University, 3-1-1 Maidashi, Higashi-ku, Fukuoka City, 812-8582 Japan; 2 Department of Anatomic Pathology, Pathological Sciences, Graduate School of Medical Sciences, Kyushu University, Fukuoka, Japan; 3 Department of Orthopedic Surgery, National Hospital Organization Kyushu Medical Center, Fukuoka, Japan; 4 Department of Musculoskeletal Oncology and Rehabilitation, National Cancer Center, Tokyo, Japan; 5 Department of Orthopaedic Surgery, Kyushu Rosai Hospital, Fukuoka, Japan

**Keywords:** Biochemistry, Cell biology, Molecular biology, Biomarkers, Medical research

## Abstract

Denosumab is a game-changing drug for giant cell tumor of bone (GCTB); however, its clinical biomarker regarding tumor ossification of GCTB has not been elucidated. In this study, we investigated the relationship between Wnt/β-catenin signaling and the ossification of GCTB and evaluated whether endogenous nuclear β-catenin expression predicted denosumab-induced bone formation in GCTB. Genuine patient-derived primary GCTB tumor stromal cells exhibited osteoblastic characteristics. Identified osteoblastic markers and nuclear β-catenin translocation were significantly upregulated via differentiation induction and were inhibited by treating with Wnt signaling inhibitor, GGTI-286, or selective Rac1-LEF inhibitor, NSC23766. Furthermore, we reviewed the endogenous ossification and nuclear β-catenin translocation of 86 GCTB clinical samples and elucidated that intra-tumoral ossification was significantly associated with the nuclear translocation. Three-dimensional quantitative analyses (n = 13) of tumoral CT images have revealed that the nuclear β-catenin translocation of naïve GCTB samples was significantly involved with the denosumab-induced tumor ossification. Our findings suggest a close relationship between the nuclear β-catenin translocation and the osteoblastic differentiation of GCTB. Investigations of the nuclear β-catenin in naïve GCTB samples may provide a promising biomarker for predicting the ossification of GCTB following denosumab treatment.

## Introduction

Giant cell tumor of bone (GCTB) is a bone neoplasm characterized by locally aggressive and massive bone destruction ^1–4^. The chief components of GCTB are osteoclast-like giant cells (GCTB-OCs) that express RANK and fibroblast-like spindle stromal cells that express RANKL, a key mediator of osteoclast activation ^5–8^. Recently, Histone 3.3 G34W (H3G34W), a driver mutation in H3 histone family member 3A (encoded by *H3F3A*), was identified as a specific surrogate marker of GCTB ^6,9,10^. The presence of this mutation confirmed that GCTB stromal cells (GCTB-SCs) are the actual neoplastic component of this tumor ^11^.

Surgical resection has been the treatment of choice for GCTB ^4,12–14^. However, curative resection for GCTB in the axial skeleton (spine, sacrum, and pelvis) is not often feasible ^4,12,14^. Denosumab, a fully human monoclonal antibody that inhibits RANKL, was recently approved for the treatment of GCTB ^15–18^. Denosumab inhibits RANKL, thereby preventing RANK–RANKL interactions, resulting in deletion of GCTB-OCs and decreasing tumor-induced osteolysis ^15–18^. In addition, diffuse bone formation with the peripheral sclerotic rim is often observed after denosumab treatment ^4^. These histological and clinical consequences can result in a more comfortable operative procedure and decrease surgical morbidity ^4,15–17^. However, the underlying mechanism of that ossification was not known.

Although osteolysis is the characteristic feature of GCTB, intra-tumoral and peripheral bone develops in 30–50% of cases of GCTB ^19,20^. Consistent with this observation, several previous studies reported that GCTB-SCs have the ability to undergo osteoblastic differentiation ^1,7,8,13,21,22^. One essential requirement for osteoblastic differentiation could be the association between WNT/β-catenin signaling and its target gene, Runt-related transcription factor2 (*RUNX2*) ^23–25^. Previous studies reported nuclear β-catenin translocation and expression of RUNX2 in GCTB-SCs ^21,26^. These results suggest the involvement of the so-called "canonical" Wnt signaling pathway in the osteoblastic differentiation of GCTB-SCs. However, this hypothesis has remained unproven ^7^, and no useful markers have been identified to predict bone formation in GCTB.

In this study, we investigated the relationship between the Wnt/β-catenin signaling, a critical pathway for osteogenic differentiation, and ossification of GCTB using patient-derived primary cultures. In addition, we investigated whether nuclear β-catenin translocation in GCTB-SCs predicted bone formation after denosumab treatment.

## Results

### Denosumab induced bone formation in the patient with GCTB

**Figure 1:**
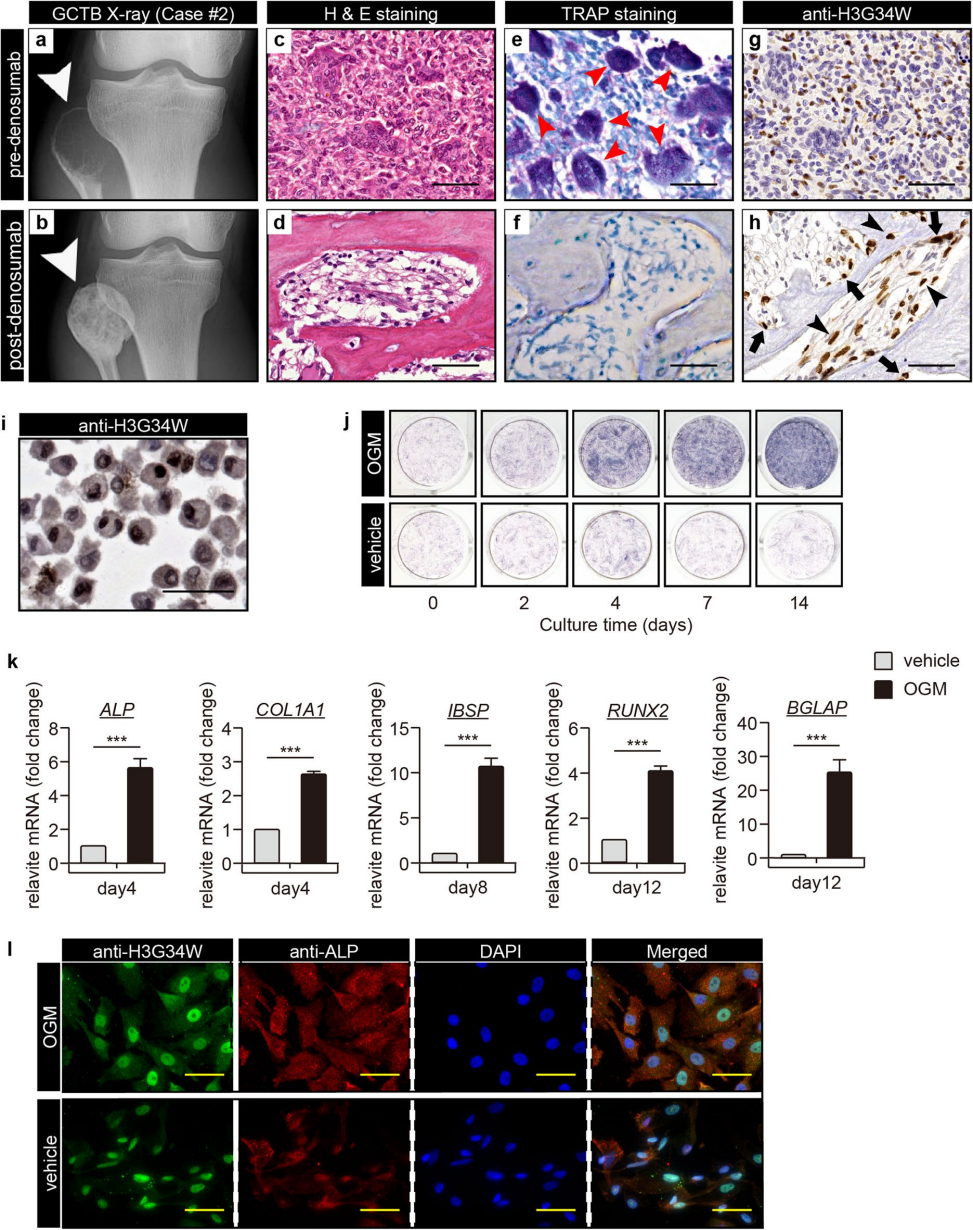
GCTB stromal cells can differentiate into bone. (**a**) A radiograph of the affected bone lesion in a patient with GCTB (case #2 in Table 1) shows osteolysis of the right proximal fibula (arrowhead) before denosumab treatment. (**b**) Distinctive skeletogenesis in the same case (arrowhead) after denosumab treatment. (**c**–**h**) Multinucleated giant cells were surrounded by abundant mononuclear and anti-Histone 3.3 G34W (H3G34W)-positive GCTB stromal cells (GCTB-SCs). With denosumab administration, multinucleated giant cells (**e,** red arrow) were reduced and osteoid appeared with the remaining tumor cells (**d**, **f**, **h**). Anti-H3G34W-positive GCTB-SCs remained adjacent to (arrow) or inside (arrowhead) the osteoid (**g**, **h**). Scale bar, 50 μm. (**i**) Established primary cultures of GCTB-SCs (pGCTB-SCs) were diffusely positive for the H3G34W mutation. Scale bar, 50 μm. (**j**) Cytochemical staining for alkaline phosphatase (ALP) activity. pGCTB-SCs were cultured in the presence or absence of osteogenic medium (OGM) for the indicated periods. The induction of osteoblast differentiation increased ALP activity in a time-dependent manner. (**k**) pGCTB-SCs exhibited osteoblastic characteristics by differentiation induction. Cells were cultured for the indicated times in OGM, and mRNA expressions of osteoblastic markers, *ALP*, *COL1A1*, *IBSP* (bone sialoprotein), *RUNX2,* and *BGLAP* (osteocalcin) were measured by qRT-PCR. Gene expression at each stage is given relative to the level of vehicle (as control). Values represent means ± SD (n = 4). *COL1A1*, collagen type I alpha 1; *IBSP*; integrin-binding sialoprotein; *RUNX2*, runt-related transcription factor-2; *BGLAP*, bone gamma-carboxyglutamate protein. ^***^P < 0.001 (**l**) H3G34W-positive tumor cells retained the ability to differentiate into osteoblasts. Cells were maintained with or without OGM for six days and then double-stained with anti-H3G34W and anti-ALP antibodies. Fluorescence images were observed under a confocal microscope: scale bars, 50 μm.

In patients with GCTB, administration of denosumab often induced massive bone formation, as shown in Fig. 1a and b. Histologically, we observed loss of GCTB-OCs with marked bone formation (Fig. 1c–f). Meanwhile, H3G34W-positive GCTB-SCs remained adjacent to or within the newly formed bone (Fig. 1g, h and S1), suggesting that GCTB-SCs were of the osteoblast lineage, as previously reported ^1,5,27^.

### Isolation of GCTB-SCs and induction of bone differentiation

To assess the osteogenic potential of GCTB-SCs, we harvested primary cultures from freshly sorted samples (pGCTB-SCs). As shown in Fig. 1i, almost all pGCTB-SCs were positive for H3G34W, demonstrating that the cultures were pure. Next, we grew pGCTB-SCs in the presence or absence of OGM. The presence of OGM strongly stimulated ALP expression in pGCTB-SCs in a time-dependent manner (Fig. 1j and S2a). In addition, the mRNA levels for representative osteoblast genes *ALP*, *COL1A1*, *IBSP* (bone sialoprotein), *RUNX2,* and *BGLAP* (osteocalcin) were significantly increased by the presence of OGM (Fig. 1k) in a time-dependent manner (Fig. S2b). Osteoblastic differentiation of pGCTB-SC was not affected by denosumab treatment (Figs. S2c,S2d). Fluorescence immunocytochemical analysis revealed that H3G34W-positive pGCTB-SCs expressed high levels of ALP after OGM treatment (Fig. 1l). Based on these findings, we confirmed that H3G34W-positive pGCTB-SCs had the capacity to differentiate into bone-forming osteoblasts.

### Effect of the activation of Wnt/β-catenin signaling on osteoblastic differentiation of pGCTB-SCs

**Figure 2:**
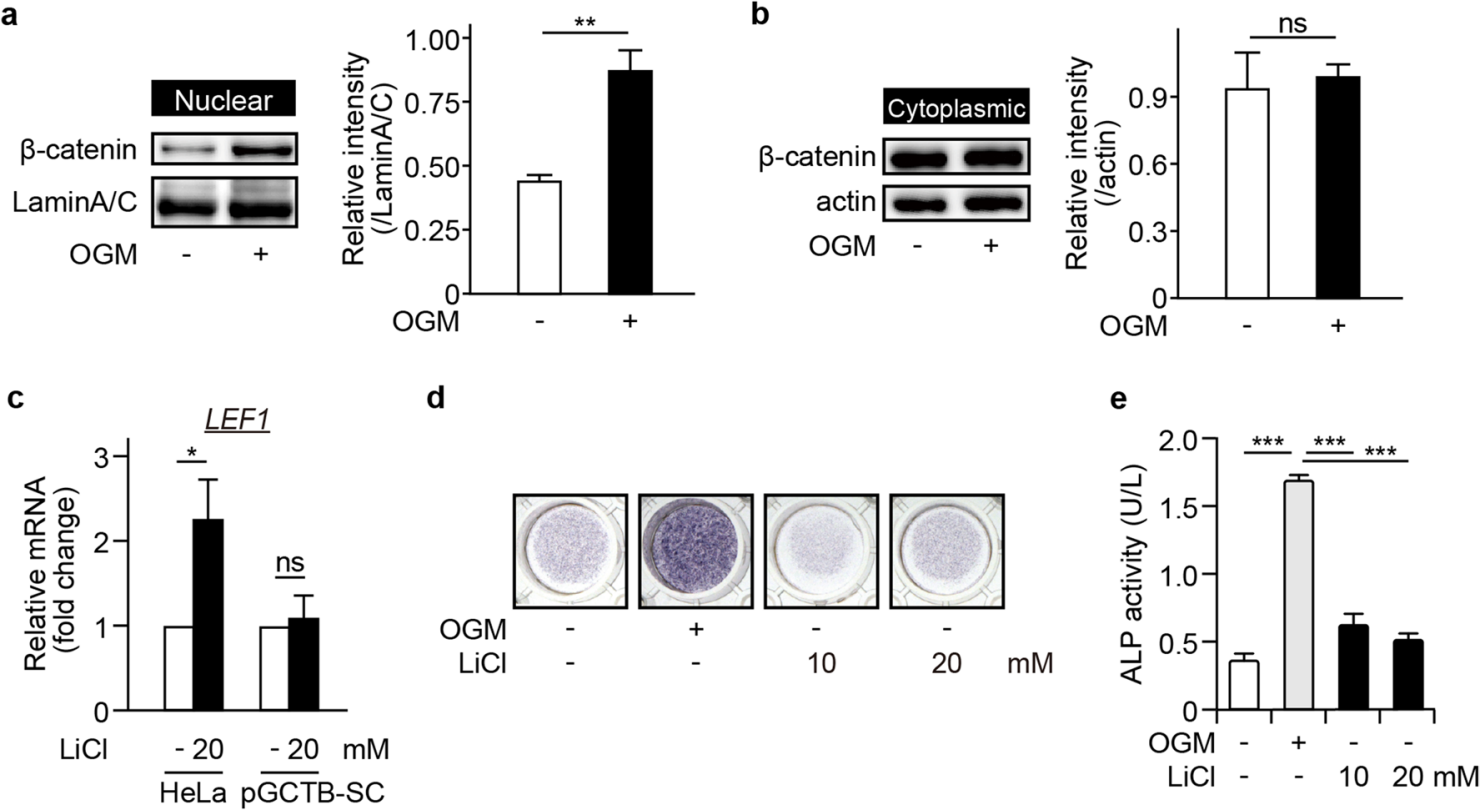
Nuclear β-catenin translocation (nuclear β-catenin translocation) is essential but not sufficient for bone differentiation of GCTB. (**a**, **b**) Induction of bone differentiation increased nuclear β-catenin translocation. pGCTB-SCs were treated with or without OGM for 12 h, and β-catenin was analyzed in the nuclear (**a**) and cytoplasmic (**b**) fractions by western blotting. Lamin A/C was adopted as a loading control for nuclear extracts and actin for cytoplasmic extracts. Experiments were repeated three times, and representative images were shown. Relative intensity to each internal control was calculated. Values represent means ± SD. ^**^P = 0.006. ns, not significant. (**c**) LiCl, a canonical Wnt agonist, did not induced LEF1, a gene involved in canonical Wnt pathway. HeLa was used as positive control for LiCl administration. Values represent means ± SD (n = 3). *P = 0.042. ns, not significant. (**d**, **e**) LiCl did not induced ALP expression in pGCTB-SCs. Tumor cells were cultured with 10 or 20 mM LiCl for 1 week, and then cytochemical staining was performed (**d**). Effects of LiCl on ALP expression of pGCTB-SCs were also evaluated by measuring OD_405_ (**e**). Values represent means ± SD (n = 4). ^***^P < 0.001.

The osteoblastic differentiation from mesenchymal precursors is regulated by the Wnt and BMP signaling pathways ^23,28–30^. In addition, previous studies reported nuclear β-catenin translocation in GCTB-SCs ^31^. Hence, we hypothesized that the canonical Wnt pathway regulates the osteoblastic differentiation of pGCTB-SCs. Accordingly, we grew pGCTB-SCs in OGM, lysed the cells, and purified cytoplasmic and nuclear protein fractions. By western blotting, we confirmed that the nuclear β-catenin translocation was upregulated within 12 h after OGM treatment, although cytoplasmic β-catenin remained unchanged (Fig. 2a, b and S5). To further determine whether the nuclear β-catenin translocation was due to activation of the canonical Wnt pathway, we treated pGCTB-SCs with LiCl which activates canonical Wnt signaling ^7^. Interestingly, treatment with LiCl induced neither LEF1, a gene involved in the canonical Wnt/β-catenin signaling pathway, nor ALP expression in pGCTB-SCs (Fig. 2c–e).

### Inhibition of the nuclear β-catenin translocation by geranylgeranyltransferase inhibitor abolished osteoblastic differentiation of pGCTB-SCs

**Figure 3:**
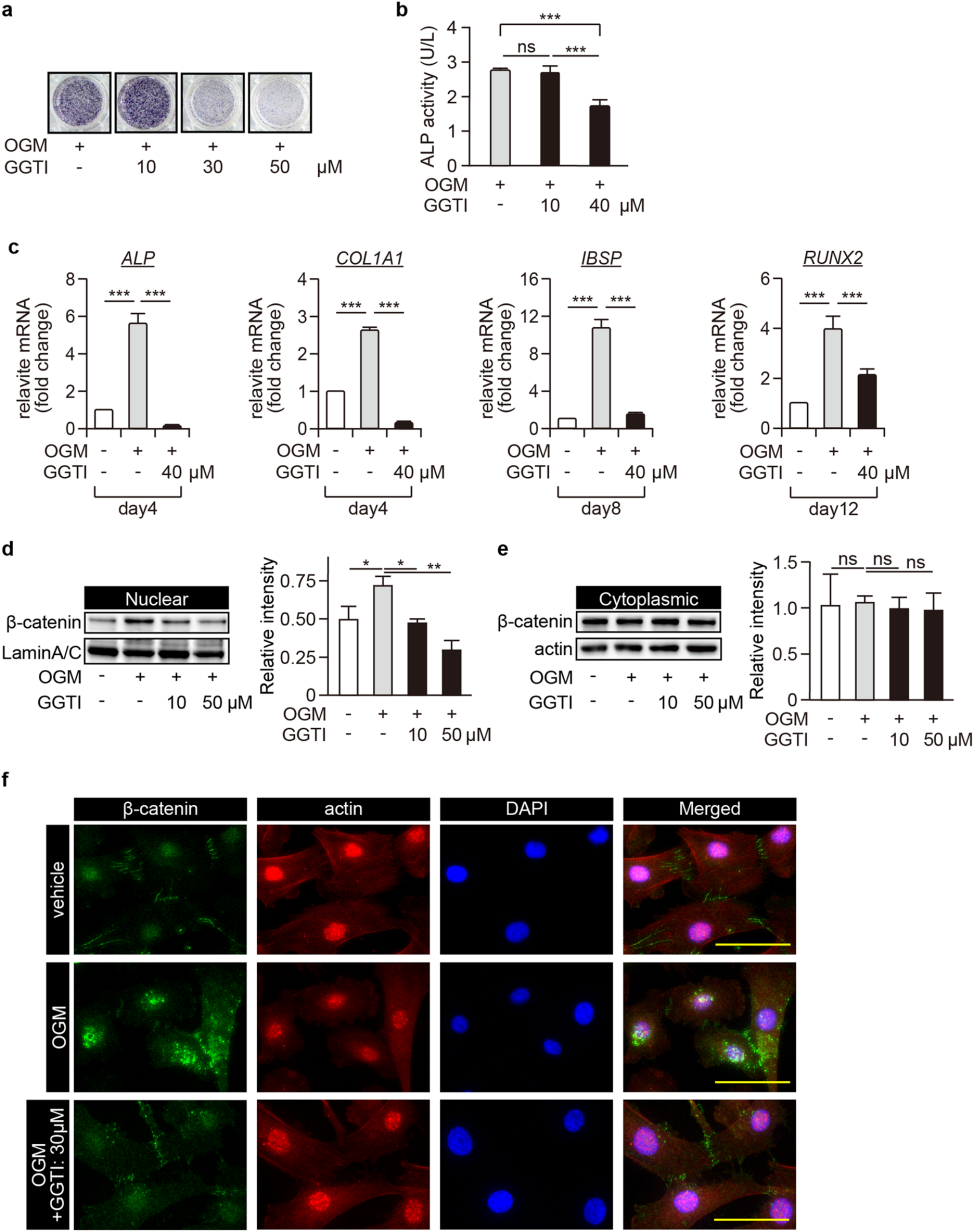
Inhibition of nuclear β-catenin translocation abolishes osteoblastic differentiation of pGCTB-SCs. (**a**) GGTI-286 (GGTI), a chemical inhibitor of geranylgeranyltransferase, decreased ALP expression levels in a concentration-dependent manner. pGCTB-SCs were differentiated in OGM with or without GGTI-286 for 6 days, and then ALP staining was performed. DMSO was used as a control. (**b**) pGCTB-SCs were cultured with or without 10 μM or 40 μM GGTI for 6 days, and ALP activity was determined. Each measurement was normalized according to the standard curve. Values represent means ± SD (n = 4). ***P < 0.001. ns, not significant. (**c**) GGTI diminished expression of osteoblastic markers, *ALP*, *COL1A1*, *IBSP,* and *RUNX2*. Cells were cultured with or without 40 μM GGTI for the indicated periods, and the mRNA levels were investigated by qRT-PCR. Gene expression at each stage is given relative to the level in the OGM-free control. Values represent means ± SD (n = 4). ***P < 0.001. (**d**, **e**) GGTI reduced OGM-induced nuclear β-catenin translocation. pGCTB-SCs were pre-treated with 10 μM or 50 μM GGTI-286 for 12 h and further incubated with OGM for 12 h. β-catenin was analyzed in the nuclear (**d**) and cytoplasmic (**e**) fractions by western blotting. Experiments were repeated three times, and representative images were shown. Relative intensity to each internal control was calculated. Values represent means ± SD. *P < 0.05. **P < 0.01. ns, not significant. (**f**) Nuclear β-catenin translocation was increased by induction of differentiation and abolished by GGTI, although the accumulation of β-catenin involved in cell–cell adhesion was not inhibited. pGCTB-SCs were cultured in OGM with 30 μM GGTI for 12 h, and then stained with anti-β-catenin and anti-actin antibodies. Fluorescence images were observed under a confocal microscope: scale bars, 50 μm.

Next, we studied the effect of GGTI-286 (GGTI), a recently identified Wnt/β-catenin pathway inhibitor ^32^, on the osteoblastic differentiation of pGCTB-SCs. GGTI dramatically decreased OGM-induced ALP expression in pGCTB-SCs in a dose-dependent manner (Fig. 3a and b). GGTI also significantly abolished the osteoblastic makers' expression (*ALP*, *COL1A1*, *IBSP*, and *RUNX2*; Fig. 3c). We also confirmed that GGTI inhibited the OGM-induced nuclear β-catenin translocation in a dose-dependent manner whereas GGTI did not affected on cytoplasmic β-catenin (Fig. 3d, e and S6). The immunocytochemical analysis also revealed that GGTI abrogated the OGM-induced nuclear β-catenin translocation in pGCTB-SCs (Fig. 3f). These results suggested that the nuclear β-catenin translocation was indispensable for osteoblastic differentiation of pGCTB-SCs.

### Activation of Rac1 was required for osteoblastic differentiation of pGCTB-SCs

**Figure 4:**
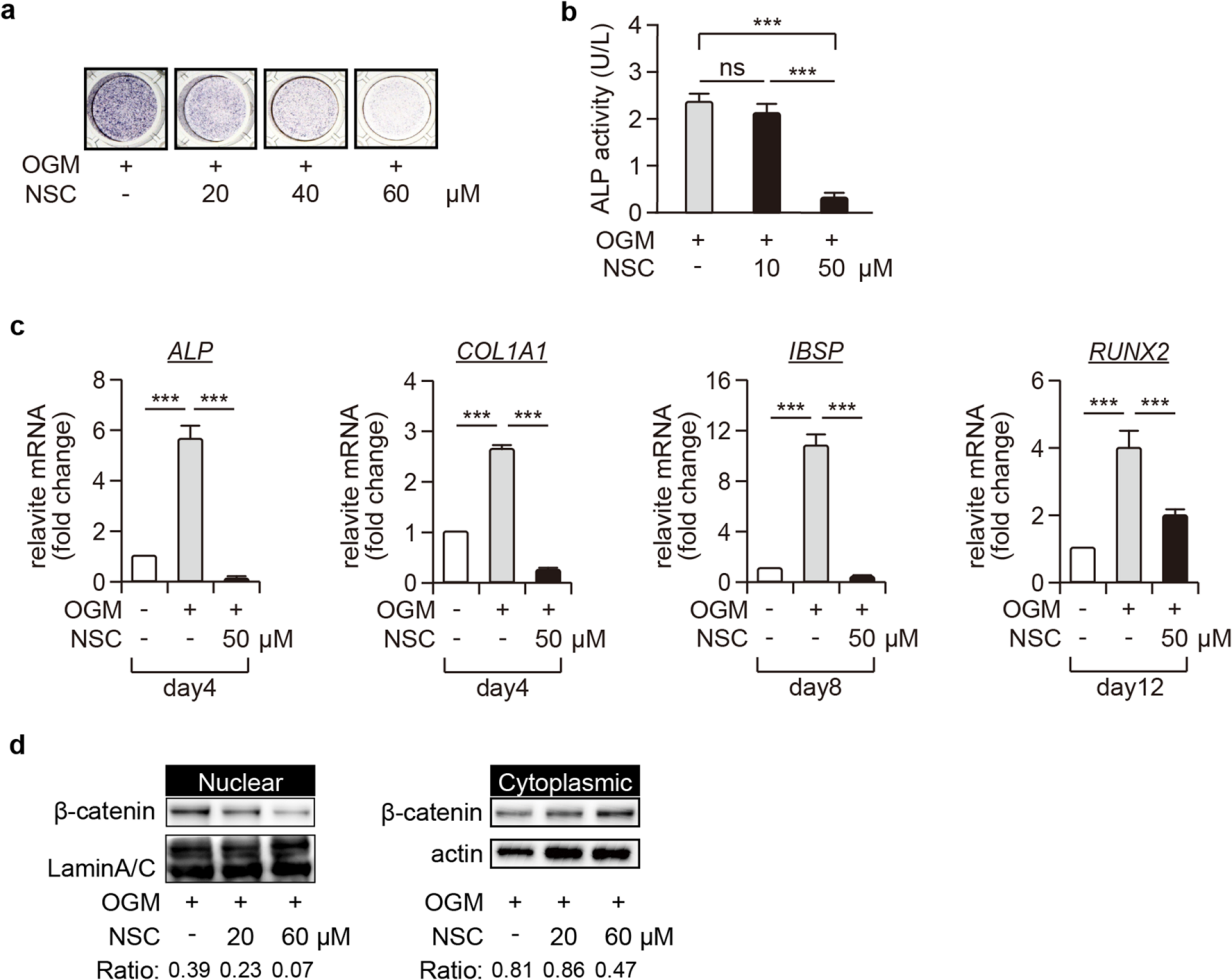
Inhibition of RAC1 disrupts nuclear β-catenin translocation and osteogenic differentiation of pGCTB-SCs. (**a**) NSC23766 (NSC), a selective RAC1-GEF inhibitor, decreased ALP expression levels in GCTSCs in a concentration-dependent manner. pGCTB-SCs were differentiated in OGM with or without NSC for 6 days, and ALP staining was subsequently performed. (**b**) pGCTB-SCs were cultured with or without 10 μM or 50 μM NSC for 6 days, and ALP activity was determined by measuring OD_405_. Values represent means ± SD (n = 4). ^***^P < 0.001. ns, not significant. (**c**) NSC also diminished expression of osteoblastic markers, *ALP*, *COL1A1*, *IBSP,* and *RUNX2*. Cells were cultured with or without 50 μM NSC for the indicated periods, and the mRNA levels were investigated by qRT-PCR. Values represent means ± SD (n = 4). ^***^P < 0.001. (**d**) NSC decreased the OGM-induced nuclear β-catenin translocation. pGCTB-SCs were pre-treated with 20 μM or 60 μM NSC for 12 h and further incubated with OGM for 12 h. β-catenin was analyzed in the nuclear and cytoplasmic fractions by western blotting. Experiments were repeated three times, and representative images were shown.

We then investigated the underlying mechanism by which GGTI inhibited the OGM-induced nuclear β-catenin translocation. A previous study claimed that GGTI binds a geranylgeranyl group on the C terminus of Rho family small GTPases, including Rac1 ^33^. Furthermore, Rac1 promotes the nuclear β-catenin translocation through phosphorylation at Ser191 by a downstream effector kinase, JNK ^34^. Hence, we analyzed the effect of a selective Rac1-GEF inhibitor, NSC23766 (NSC), on OGM-induced osteoblastic differentiation of pGCTB-SCs. Like GGTI, NSC also decreased the expression levels of ALP and other osteoblastic markers in pGCTB-SCs following OGM treatment (Fig. 4a–c). NSC also diminished nuclear β-catenin translocation following OGM treatment (Fig. 4d, S3 and S7). Together, these findings indicate that activation of Rac1 is required for the nuclear β-catenin translocation and subsequent osteoblastic differentiation of pGCTB-SCs.

### Nuclear β-catenin translocation in GCTB-SCs of patients with GCTB and its association with intra-tumoral ossification of GCTB

**Figure 5:**
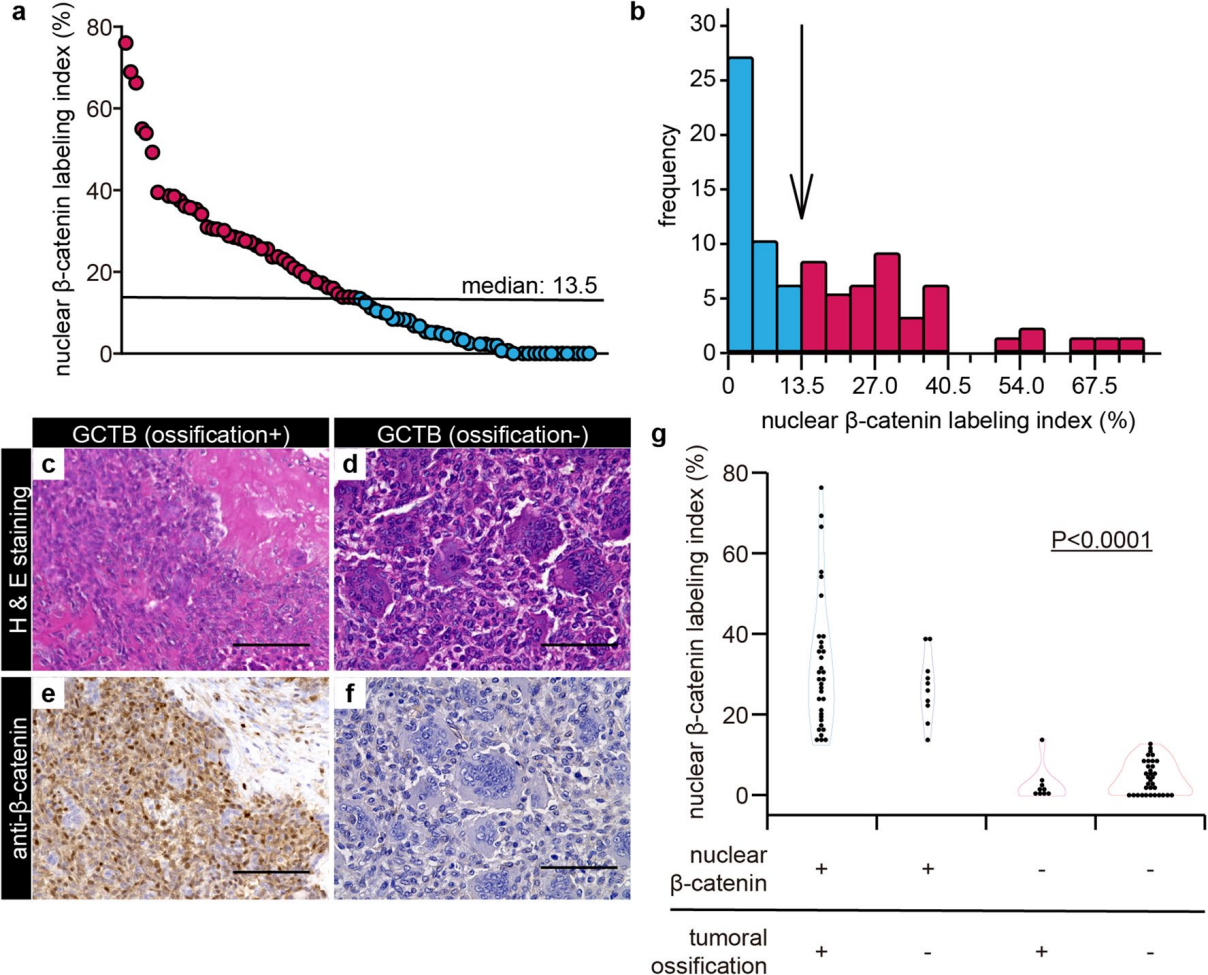
Nuclear β-catenin translocation in GCTB-SCs was significantly associated with endogenous ossification of GCTB. (**a**) Eighty-six clinical GCTB samples were retrospectively reviewed, and the nuclear β-catenin labeling index (NBLI) in the GCTB-SCs was calculated. Three to five hotspots per high-power field were evaluated, depending on the level of nuclear β-catenin expression. The median value (13.5) was set as a cut-off for NBLI. (**b**) Histogram showing non-normal distribution of NBLI in the GCTB-SCs. The image is color-coded according to the median value of NBLI (arrow). (**c**–**f**) Representative images of GCTB specimens with or without intra-tumoral ossification and the results of immunohistochemistry for β-catenin. A typical massive intra-tumoral ossification with focal tumor region was strongly positive for nuclear β-catenin in the stromal component with the ossified case, whereas osteoclastic giant cells were negative for that (**c**, **e**). The stromal components surrounding the remaining giant cells were negative for β-catenin in the non-ossified case (**d**, **f**). Scale bar, 100 μm. (**g**) Violin plots of NBLI of GCTB-SCs depicting the association between the nuclear translocation of β-catenin with intra-tumoral ossification of GCTB. The nuclear β-catenin–positive group exhibited a significantly higher intra-tumoral ossification rate than the negative group (P < 0.001, Fisher’s exact test).

Some cases of GCTB exhibit spontaneous intra-tumoral ossification, whereas others do not ^20,35^. Based on our in vitro results, we hypothesized that in cases in which endogenous nuclear β-catenin translocation occurs in GCTB-SCs, the tumor cells might be more likely to differentiate into osteoblasts. To test this notion, we first assessed the distribution of nuclear β-catenin labeling index (NBLI), a method to evaluate nuclear β-catenin ^46,47^, in GCTB-SCs using 91 clinical samples of GCTB. Of those samples, three cases were excluded due to overlap, one was removed due to low sample quantity, and another was excluded because the associated patient data were not available. Ultimately, a total of 86 tumor sections were retrospectively reviewed. The background data on these sections are presented in Supplementary Table S1. A retrospective evaluation revealed that the distribution of NBLI in GCTB-SCs was variable, ranging from 0% to 76.4% (median: 13.5%, Fig. 5a). In addition, the histogram of NBLI in GCTB-SCs exhibited a non-normal distribution (Fig. 5b). We set the median as the cut-off value of the NBLI and subdivided the cases of GCTB into two groups as follows: positive (NBLI > 13.5%, positive group, n = 43) and negative for nuclear β-catenin translocation (NBLI ≤ 13.5%, negative group, n = 43). Retrospective evaluation of NBLI significantly coincided among reviewers (R^2^ = 0.877 by Pearson product-moment correlation coefficient; P < 0.001, Fig. S4) When we examined the prevalence of intra-tumoral ossification of GCTB, we found that 42 out of 86 samples (48.8%) developed intra-tumoral ossification (Fig. 5c and d), consistent with a previous report ^20^. Remarkably, the positive group exhibited a significantly higher rate of intra-tumoral ossification (Fig. 5e) than the negative group (Fig. 5f), as shown in Fig. 5g (*P* < 0.001, Fisher’s exact test).

### The number of GCTB-SCs with nuclear β-catenin translocation in biopsy samples was correlated with the degree of bone formation after denosumab treatment

The diversity of endogenous nuclear β-catenin translocation in GCTB-SCs may explain differences in bone formation after denosumab treatment. To address this issue, we studied 14 consecutive cases of GCTB that received denosumab. One case was excluded due to a problem with the specimen; the remaining 13 tumor samples were reviewed. Detailed information on individual cases is summarised in Table 1.

**Table 1:** Patient characteristics, with distribution of nuclear β-catenin and quantification of tumor ossification.

#	Age/Gender	Site	Campanacci grade	NBLI (%)	Agatston score^a^		
					Entire	Peripheral	Intra-tumor
1	68/F	Rt. tibia	II	76.4	4.75	2.27	17.36
2	20/F	Rt. fibula	II	69.4	7.90	4.68	14.55
3	28/M	Lt. femur	II	38.7	2.44	2.24	3.93
4	41/F	C-spine	II	30.7	5.41	8.50	3.01
5	31/M	Rt. femur	II	23.9	67.79	75.19	62.60
6	40/M	L/E	N/A	18.9	12.42	26.73	6.35
7,	84/F	Rt. humerus	III	18.6	3.73	1.99	9.36
8	74/F	Lt. tibia	III	12.6	1.06	1.04	1.17
9	27/F	Sacrum	III	10.1	1.31	1.45	1.05
10	35/M	Rt. humerus	III	8.5	1.55	1.20	6.52
11	39/F	Rt. femur	III	0.0	1.20	1.16	1.33
12	66/M	Rt. tibia	II	0.0	1.30	1.29	1.30
13	18/M	Rt. humerus	II	0.0	1.37	1.15	3.05

*C-spine* cervical spine; *L/E* lower extremity (Soft tissue recurrence); *Lt* left; *NBLI* Nuclear β-catenin labeling index; *N/A* not acquired; *Rt* right.

^a^Each Agatston score of post-denosumab treatment was normalized against the pre-treatment score.

**Figure 6:**
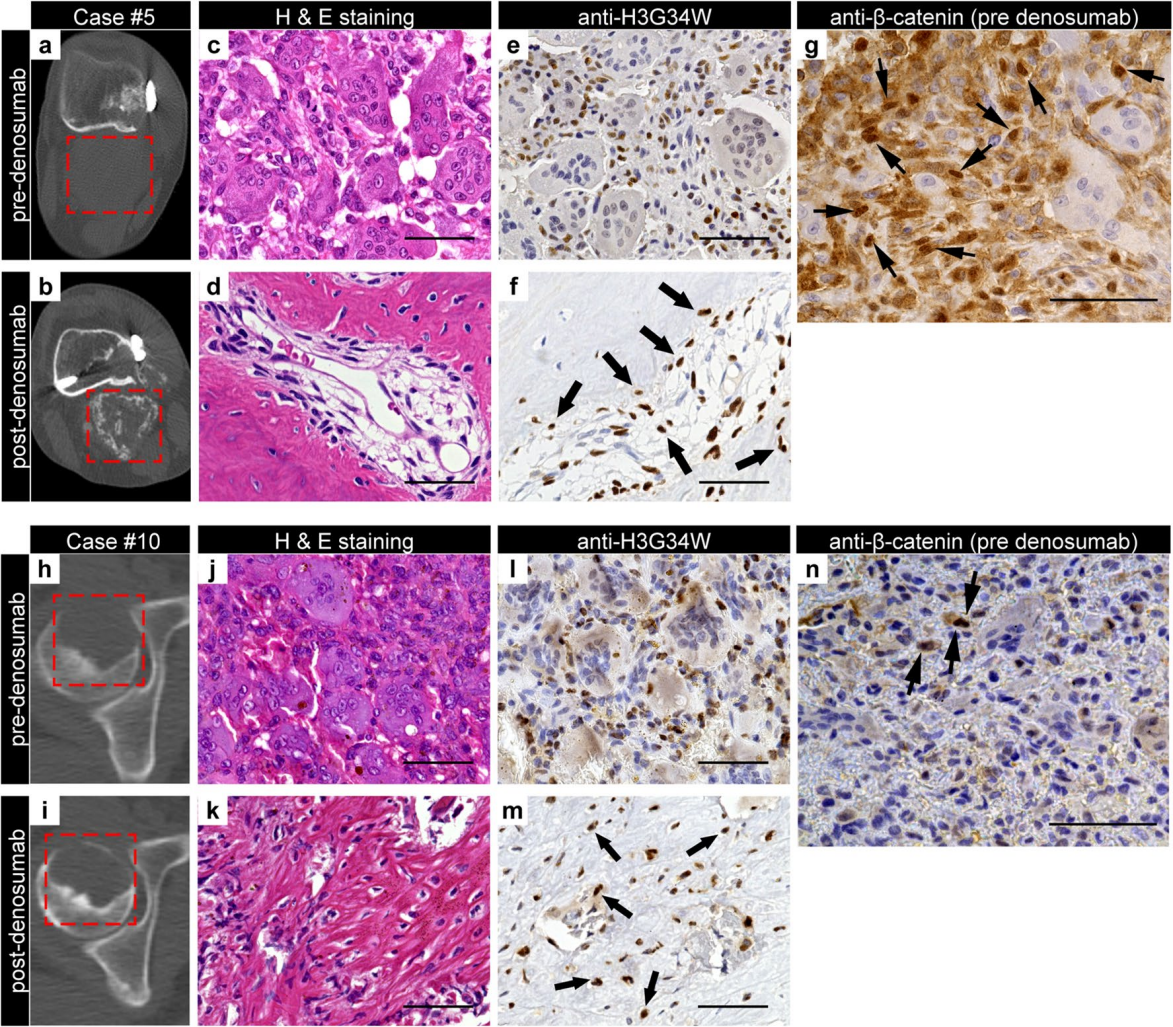
Nuclear positivity of β-catenin in GCTB-SCs predicts intra-tumoral bone formation of GCTB after denosumab treatment. (**a**, **b**) Axial computed tomography (CT) images (case #5) with no ossification before denosumab treatment (**a**) and massive bone formation after denosumab treatment (**b**). The red dashed line represents a tumor area located dorsal to the femoral bone. (**c**, **d**) Osteoclastic giant cells disappeared after denosumab treatment, and significant bone formation with residual focal tumor lesions was observed. (**e**, **f**) Mononuclear stromal cells were diffusely positive for the H3G34W mutation, whereas osteoclastic giant cells were negative. Transformed cells after denosumab treatment were positively stained for the H3G34W mutation (arrow). (**g**) A denosumab-naïve tumor specimen exhibited strong nuclear positivity for β-catenin (arrowhead) in GCTB-SCs, whereas giant cells were negative. (**h**, **i**) Another axial CT image (case #10) with an osteolytic lesion in the right femoral head (**h**). Bone formation was scarce despite several administrations of denosumab (**i**). The red dashed line represents tumor lesions. (**j**, **k**) Osteoclastic giant cells disappeared after denosumab treatment (**j**); however, the specimen consisted mainly of fine reticular fibrosis (**k**). (**l**, **m**) Mononuclear cells before denosumab treatment (**l**) and transformed cells inside fibrosis after that (**m**) were positive for H3G34W. (**n**) Stromal tumor cells were weakly positive for nuclear β-catenin in a poorly ossifying case (arrowhead): scale bars, 50 μm.

First, we examined NBLI in GCTB-SC using naïve GCTB samples prior to denosumab administration. Seven cases were positive and six were negative. Case #5, a positive group representative, exhibited massive bone formation after denosumab treatment, as revealed in CT images (Fig. 6a and b). Histologically, deletion of osteoclastic giant cells and prominent bone formation was observed (Fig. 6c and d). However, H3G34W-positive GCTB-SCs were still present in the specimen (Fig. 6e and f). Biopsy samples acquired before denosumab treatment revealed that multiple GCTB-SCs were positive for nuclear β-catenin translocation (NBLI = 23.9, Fig. 6g). Meanwhile, in case #10, a typical case of the negative group, bone formation after denosumab treatment was scarce (Fig. 6h and i). Additionally, the surgically resected samples exhibited loss of osteoclast-like giant cells but not bone formation (Fig. 6j and k). However, stromal cells were positive for H3G34W, as were the biopsy samples (Fig. 6l and m). Importantly, before denosumab treatment, only a few GCTB-SCs were positive for nuclear β-catenin translocation (NBLI = 8.5, Fig. 6n).

**Figure 7:**
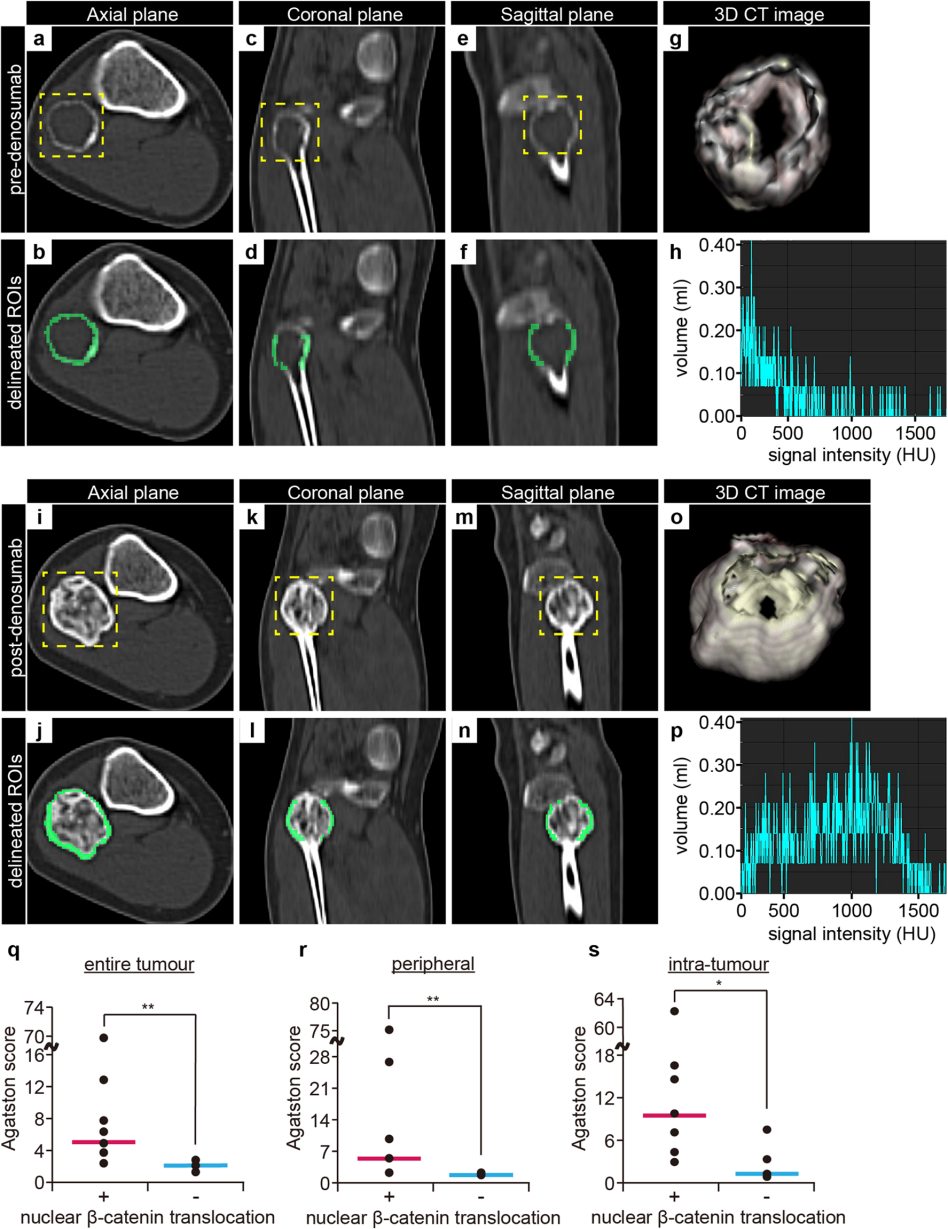
Nuclear β-catenin positivity of GCTB-SCs is significantly associated with bone formation after denosumab treatment. To elucidate the relationship between β-catenin stainability and tumoral ossification, we performed quantitative evaluations using SYNAPSE VINCENT. (**a**–**g**) Representative images of three-dimensional analysis before denosumab treatment (case #2). ROI in the axial (**a**, **b**), coronal (**c**, **d**), and sagittal (**e**, **f**) CT images were delineated, and the 3D tumor model was automatically depicted (**g**). (**h**) Histogram image of the ROI before denosumab treatment. Tissue volumes with more than 130 HU were identified as significant calcifications, and Agatston scoring was performed as previously described^27, 28^. (**i**–**n**) Representative three-dimensional image after the treatment. ROI in the axial (**i**, **j**), coronal (**k**, **l**), and sagittal (**m**, **n**) CT images were used for 3D model analysis (**o**). Bright yellow lines represent the peripheral rim of the ROI. (**p**) Histogram image of the ROI after denosumab treatment. In the ossified case, the histogram image was leptokurtic, and the degree of skew was significant. (**q**–**s**) The relationship between nuclear β-catenin stainability and intra-tumoral ossification was evaluated using Agatston score (n = 13). The stainability was significantly associated with the score in the entire (**q**), peripheral (**r**), and intra-tumoral areas (**s**) (^**^P = 0.003 for entire and peripheral tumor, and ^*^P = 0.02 for intra-tumor of GCTB). Each post-treatment Agatston score was normalized against the corresponding pre-denosumab score.

We next quantified denosumab-induced bone formation using CT images, as described previously ^36^. In the window setting where the tumor margins were discernible, ROIs for histogram analyses were semi-automatically delineated by tracing the tumors' outer margins before (Fig. 7a–g) and after (Fig. 7i–o) denosumab treatment. The entire, peripheral, and intra-tumor Agatston scores of the GCTBs were obtained by histogram analyses using SYNAPSE VINCENT (Fig. 7h and p). Notably, after denosumab administration, the positive group exhibited significantly higher ossification than the negative group (Fig. 7q–s). Therefore, the positive group would have more chances to undergo denosumab-induced bone formation. Thus, the NBLI in GCTB-SCs in biopsy samples represents a reasonable and straightforward biomarker for predicting the degree of bone formation after denosumab treatment.

## Discussion

The introduction of denosumab has attracted attention as a novel therapy of GCTB. Several clinical studies have confirmed that denosumab administration prevents osteolysis, concomitant with the deletion of GCTB-OCs ^15,16,18,37^. It may also cause additional histological consequences, including central sclerosis with peripheral bone formation, enabling surgical downstaging. However, the degree of bone formation is case-dependent, and we experienced some cases with little bone formation despite the treatment. Therefore, we assumed that predicting subsequent bone formation after denosumab administration would have clinical benefits for decision making, enabling clinicians to achieve optimal treatment for GCTB.

We first focused on the mechanism of osteoblastic differentiation of GCTB-SCs. H3G34W-positive GCTB-SCs had the ability to differentiate into osteoblasts but not chondrocytes or adipocytes (data not shown), indicating that GCTB-SCs did not retain pluripotency. During osteoblastic differentiation, the canonical Wnt/β-catenin signaling is the dominant pathway ^26^. However, in our study, forced activation of LRP- and GSK3β-mediated canonical Wnt/β-catenin signaling did not cause differentiation. By contrast, our results showed that the apparent nuclear β-catenin translocation was associated with the osteoblastic differentiation of GCTB-SCs and that a recently identified inhibitor of the canonical Wnt signaling, GGTI, effectively inhibited it.

GGTI inhibits protein prenylation, and this process is essential for the correct localization and functions of GTPases, including Rac1 ^32^. In addition, Rac1 stimulates nuclear β-catenin translocation through phosphorylation at Ser191 by a downstream effector kinase, JNK ^27^. Consistent with these previous results, we found that Rac1 inhibition suppressed nuclear β-catenin translocation and osteoblastic differentiation of GCTB-SCs. Therefore, we considered that denosumab administration caused activation of Rac1 and triggered the nuclear localization of β-catenin, followed by osteoblastic differentiation of GCTB-SCs, ultimately resulting in the cessation of tumor activity. If this is the case, forced activation of Rac1 in GCTB-SCs by a potent Rac1-agonist, such as the recently discovered natural polyketide deacetylmycoepoxydiene ^38^, may stimulate the osteoblastic differentiation of GCTB-SCs, and combination treatment with denosumab and Rac1-agonist could be an effective strategy for the treatment of GCTB.

We detected baseline nuclear β-catenin translocation in pGCTB-SCs, indicating intrinsic activation of canonical Wnt/β-catenin signaling. Interestingly, a previous study showed that miR-125a stimulates the translocation of β-catenin in GCTB-SCs through GSK3β-mediated canonical signaling, resulting in cell proliferation and tumorigenicity ^39^. However, extrinsic inhibition of GSK3β by LiCl did not play significant roles in osteoblastic differentiation of GCTB-SCs, as shown in Fig. 2c and d. Together, we speculated that in GCTB of naïve status, there was a baseline activation of canonical Wnt signaling that regulates the proliferation of GCTB-SCs. Meanwhile, denosumab administration may cause Rac1-associated activation of the canonical Wnt/β-catenin pathway by unknown factors.

Several stimuli can elicit Rac1 activation. For example, both Wnt-5a (a Wnt family member) and Ror2 (receptor tyrosine kinase-like orphan receptor2, a dominant receptor of Wnt-5a) activate Rac1 and induce differentiation of human mesenchymal stem cells into osteoblasts ^38,40^. Expression of the Wnt inhibitor secreted frizzled-related protein (sFRP) in GCTB-OCs has been confirmed by comprehensive mRNA profiling of GCTB-SCs ^41^. More importantly, we observed expression of Wnt-5a in GCTB-SCs (data not shown). Based on these findings, we hypothesized that deletion of GCTB-OCs by denosumab might decrease the level of sFRP in tumor tissues, activate a cascade of Wnt-5a/Ror2/Rac1 signaling, and finally cause the osteoblastic differentiation of GCTB-SCs.

As another essential feature of this study, we found that endogenous nuclear β-catenin translocation was associated with osteoblastic differentiation of GCTB-SCs via denosumab treatment. Besides, upregulated NBLI was correlated with endogenous intra-tumoral bone formation in GCTB. Notably, a recent epigenetic analysis showed that GCTB-SCs could be classified into three groups, S1 to S3. S1 cells are characterized by the expression of osteoblast-associated genes such as osteopontin, whereas S3 cells have markers of the myofibroblastic lineage, e.g., alpha-smooth muscle actin. S2 cells have features intermediate between those of S1 and S3 cells ^42^. Therefore, we speculated that GCTB-SCs with nuclear β-catenin translocation would correspond to S1 cells and be partly committed to differentiation into osteoblasts. Therefore, as discussed in a previous paragraph, the deletion of GCTB-OCs by denosumab triggered the cells' final osteoblastic differentiation. However, this notion should be further investigated.

This study had several limitations. First, the precise mechanism by which Rac1 activation occurs in GCT-SCs following denosumab treatment was not fully elucidated. More detailed in vitro experiments, including co-cultures of GCTB-SCs and GCTB-OCs, are needed to clarify this issue. Secondly, the number of patients who received denosumab treatment was relatively small in a retrospective analysis. This is because the rarity of GCTB and the recent approval of denosumab for GCTB treatment. To further validate the ability of the NBLI in GCTB-SCs to predict bone formation after denosumab treatment, we plan to conduct more extensive prospective studies in the future.

## Conclusion

In summary, our findings suggest a close relationship between the nuclear β-catenin translocation via Rac1 activation and the osteoblastic differentiation of GCTB. Investigations of NBLI in naïve GCTB samples will provide a promising biomarker for predicting the degree of bone formation after denosumab treatment.

## Materials and methods

### Reagents

Monoclonal rabbit anti-H3G34W antibody (clone RM263) was purchased from RevMab Biosciences (San Francisco, CA, USA). Monoclonal rabbit anti-β-catenin antibody (#32572), anti-alkaline phosphatase, tissue non-specific antibody (#126820), goat anti-rabbit IgG H&L preabsorbed (Alexa Fluor® 488, #150081), and goat anti-mouse IgG H&L preabsorbed (Alexa Fluor® 594, #150120) were purchased from Abcam (Cambridge, UK). Monoclonal mouse anti-actin clone C4 antibody (MAB1501) was purchased from Merck Millipore (Burlington, MA, USA). Monoclonal mouse anti-RUNX2 antibody (sc-390351) was purhchased from Santa Cruz Biotechnology (Dallas, TX, USA). A selective inhibitor of geranylgeranyltransferase1, GGTI-286 (#22756), was purchased from Cayman Chemical (Ann Arbor, MI, USA), and a selective Rac1 (a Rho-family small GTPase) inhibitor, NSC23766, was purchased from Merck Millipore.

### Immunohistochemistry for H3G34W mutation analysis

Surgically-resected GCTB clinical samples were fixed with 4% PFA and embedded in paraffin (FFPE). Immunostaining was performed as described previously ^43^. Briefly, antigen retrieval of deparaffinized, rehydrated sections was performed by heating in a microwave for 20 min with 10 mM citric acid at pH 6.0 (FUJIFILM Wako). After blocking with normal goat serum for 30 min at room temperature, the sections were incubated with anti-H3G34W monoclonal Abs (1:200) at 4 °C overnight ^44^. Endogenous peroxidase activity was blocked by incubation with 3% hydrogen peroxidase in methanol for 30 min at room temperature, and specimens were then incubated with Dako EnVision™^+^ Dual Link System-HRP (Agilent, Santa Clara, CA, USA) for 45 min at room temperature. Antibody complex was visualized using the diaminobenzidine substrate system (FUJIFILM Wako) and counterstained with hematoxylin ^44^. Section images were obtained on a Keyence BZ-X800 microscope (Keyence Corporation, Osaka, Japan), and nuclear immunoreactivity for H3G34W was evaluated.

### Tartrate-resistant acid phosphatase (TRAP) staining for GCTB-OCs

FFPE sections (not exposed to denosumab) were deparaffinized, rehydrated, and incubated with adjusted solutions for 15 min at 37℃ according to the manufacturer's protocol (TRAP/ALP staining kit, Fujifilm Wako) for the detection of TRAP activity of GTCB-OCs. The stained sections were washed three times with deionized water and counterstained with Methyl Green Solution (FUJIFILM Wako) for 5 s. Denosumab-exposed tissue samples were also stained as negative controls.

### Co-labeling assay for H3G34W and RUNX2

Adjacent pre-exposed FFPE sections were immunohistochemically stained for H3G34W and RUNX2, the master regulator of osteogenesis, to evaluate the osteogenic potential of GCTB-SCs, using ImmPRESS® Duet Double Staining Polymer Kit (Vector Laboratories, Burlingame, CA, USA). Briefly, antigen unmasking of deparaffinized, rehydrated sections was performed with citrate-based solutions at pH 6.0, and endogenous peroxidase activity was blocked by incubation with BLOXALL® Blocking Solution (Vector Laboratories) for 20 min, followed by incubation with 2.5% normal horse serum for 20 min at room temperature. The adjacent specimens were incubated with anti-H3G34W rabbit monoclonal antibody (1:200) or anti-RUNX2 mouse monoclonal antibody (1:200) at 4 °C overnight and then incubated with ImmPRESS Duet Reagent (Vector Laboratories) for 10 min at room temperature. Antibody complex was visualized by incubation with the ImmPACT DAB EqV Substrate (Vector Laboratories) for 2 min against H3G34W mutation or the ImmPACT Vector Red Substrate (Vector Laboratories) for 20 min against RUNX2.

### Establishment of primary culture and treatment

Some fresh GCTB tumor samples were obtained from surgeries and washed with warmed PBS. The samples were minced in DMEM (Thermo Fisher Scientific, Waltham, MA, USA) supplemented with 10% FBS (HyClone Laboratories, Logan, UT, USA), 100 U/ml penicillin, and 100 μg/ml streptomycin. Together with small pieces of chipped tissues, the cell suspension was transferred to culture dishes and cultured at 37 °C in a humidified atmosphere of 5% CO_2_ and 95% air. GCTB-OCs were present only in the first passage, whereas primary GCTB-SCs (pGCTB-SCs) were further amplified. Upon reaching confluence, pGCTB-SCs were sub-cultured, and the third through eighth passages were used for subsequent experiments. To induce osteogenic differentiation, confluent cells were treated with osteogenic medium (OGM) containing Minimum Essential Medium (Thermo Fisher Scientific) supplemented with 10% FBS, 100 nM dexamethasone (FUJIFILM Wako Pure Chemical Corporation, Osaka, Japan), 100 μM ascorbic acid (FUJIFILM Wako), and 10 mM β-glycerophosphoric acid (NACALAI TESQUE, INC., Kyoto, Japan) for various periods.

### Preparing cell blocks and immunocytochemistry of GCTSCs

To determine the purity of primary cultures, we prepared cell blocks and performed immunostaining with anti-H3G34W. Confluent suspensions of pGCTB-SCs were harvested, and cell pellets were prepared by centrifugation for 5 min at 1,500 rpm. The pellets were incubated overnight at 37 °C in DMEM, and then fixed for 3 h at room temperature (RT) in 10% Formalin Neutral Buffer Solution (FUJIFILM Wako). After fixation, the supernatant was aspirated, and 1% sodium alginate (FUJIFILM Wako) was added to the pellets. Gelatinous cell blocks were immediately obtained by addition of 100 μl of 1 M CaCl_2_ (FUJIFILM Wako), and the blocks were embedded in paraffin and immunohistochemically stained for H3G34W mutation.

### Alkaline phosphate staining

ALP activity is widely used to assess the early osteogenic ability of osteoblast-like cells. We seeded pGCTB-SCs into a 24-well plate at a density of 5 × 10^4^ cells per well. After 48 h of incubation, the culture medium was exchanged and further incubated for the indicated periods. The cells were washed with PBS, fixed in 10% formalin, and stained with premixed ALP substrate solutions (FUJIFILM Wako).

pGCTB-SCs were seeded into 96-well plates at a density of 5 × 10^3^ cells per well and incubated for 48 h. After the cells reached confluence, the medium was exchanged, and the samples were incubated further. ALP assays were performed using the TRACP & ALP assay kit (Takara Bio, Kusatsu, Shiga, Japan). Briefly, treated cells were lysed in extraction solution (saline with 1% NP-40), mixed with freshly prepared p-nitrophenyl phosphate substrate (12.5 mM), and incubated at 37 °C for 30 min. The optical density of p-nitrophenol at 405 nm was determined using iMark™ Microplate Absorbance Reader (Bio-Rad, Hercules, CA, USA). Finally, ALP activity was normalized with a standard curve derived from *Escherichia coli* C75 (Takara Bio).

### Quantitative real-time PCR (qRT-PCR)

Total RNA of treated pGCTB-SCs was extracted using the RNeasy Mini Kit (Qiagen, Hilden, Germany) and reverse-transcribed with PrimeScript RT Reagent Kit (Takara Bio). qRT-PCR was conducted with a LightCycler 1.5 (Perfect Real Time, Takara Bio, Kusatsu, Shiga, Japan) with the TB Green® Premix Ex Taq™ II (Takara Bio). Primers are listed in Supplementary Table S2. Data were standardized against the housekeeping gene *GAPDH*. At least four separate experiments were conducted.

### Immunofluorescence staining

pGCTB-SCs were seeded on poly-L-lysine (Fujifilm Wako)–coated cover glass at a density of 10 × 10^4^ cells and cultured for 48 h. After the cells were treated with each reagent for the indicated periods, they were fixed with 4% paraformaldehyde (FUJIFILM Wako) for 10 min at RT, permeabilized with 0.2% Triton X-100 (Sigma-Aldrich, St. Louis, MO, USA) for 15 min, and blocked with 10% goat serum (FUJIFILM Wako) for 30 min. Subsequently, the cells were incubated at 4 °C overnight with a mixture of primary antibodies diluted in 1:200 in Can Get Signal Immunostain Solution A (TOYOBO, Osaka, Japan). Samples were then washed three times with PBS and incubated with Alexa Fluor® 488 and 594 diluted in 1:200 for 1 h at RT. SlowFade Diamond antifade mountant with DAPI (Invitrogen) was used as a mounting solution. Immunostaining was visualized using fluorescence microscopy (BZ-X800; Keyence).

### Nuclear protein extraction and western blot analysis

pGCTB-SCs were seeded in 6-well dishes at a 1.2 × 10^6^ cells/well density and incubated overnight. The following day, the culture media were replaced for each reagent, and the cells were incubated for an additional 12 h. After incubation, the cells were washed twice with ice-cold PBS, scraped, and centrifuged. Cytoplasmic and nuclear proteins were isolated using nuclear and cytoplasmic extraction reagents (Thermo Fisher Scientific), to which Cell Lytic M (Sigma-Aldrich) with protease inhibitor cocktail (cOMplete™ Mini: Sigma-Aldrich) were added.

Western blotting was performed as previously described ^45^ with the following primary antibodies: β-catenin (1:1000) and actin clone C4 (1:5000), with or without rabbit polyclonal Lamin A/C antibody (1:3000, sc-20681; Santa Cruz Biotechnology). Relative intensity was calculated using the ratio of each target protein's signal intensity to internal controls' intensity, using ImageJ ver1.52p (NIH, Bethesda, MD, USA). At least three separate experiments were conducted. Full-length blots were absent because membranes were cut prior to hybridization with primary antibodies. The unedited blots including replicates were shown in Supplementary Figs. S5, S6, S7.

### Ethics and guidelines

All the methods were conducted in accordance with the Declaration of Helsinki and written informed consent was obtained from all human subjects.

### Patients and quantitative CT image analysis

To evaluate nuclear β-catenin translocation in naïve GCTB clinical samples, we performed a retrospective analysis using samples of GCTB registered in the files of the Department of Anatomic and Pathology, Graduate School of Medical Sciences, Kyushu University, Fukuoka, Japan ^44^. A total of 91 clinical samples of GCTB from 88 patients were prepared for immunohistochemistry. These tumor specimens had been acquired from biopsy or surgeries, and the existence of the H3G34W mutation had been immunohistochemically confirmed. Samples collected after denosumab treatment were excluded from the study.

Immunohistochemical staining and assessment of the nuclear β-catenin labeling index (NBLI) were performed as previously described ^46,47^. Histogram analysis was conducted to calculate the cut-off value for the NBLI. The presence of intra-tumoral ossification of GCTB was also assessed using H&E-stained sections.

Twenty-one patients were diagnosed with GCTB or received treatment for this cancer at our hospital between July 2011 and November 2020. Of those, 18 patients had received denosumab treatment (primary, n = 12; recurrent, n = 4; both, n = 2), and 16 had also undergone non-contrast CT or PET-CT evaluation before and after denosumab treatment. CT DICOM (Digital Imaging and Communications in Medicine) image datasets from identified patients were analyzed using SYNAPSE VINCENT ver6.1 (VINCENT, FUJIFILM Medical Co., Ltd.). A single musculoskeletal radiologist with eight years of experience manually delineated the regions of interest (ROI) in the axial CT images of whole slices, and three-dimensional CT images were semi-automatically acquired. Slice thickness was set at 2 or 5 mm. We identified the calcified tissue volumes (≥ 130 Hounsfield Units, HU) using VINCENT histogram analysis and quantified tumor calcification using the previously described Agatston scoring system ^36,48^. In addition, we assessed the association between nuclear β-catenin translocation and intra-tumoral ossification of GCTB.

### Statistical analysis

All experiments were repeated at least three times. Data are presented as means ± SD. Student’s t-test or Wilcoxon's rank-sum test was used for two-group comparisons. Using one-way ANOVA with the Tukey–Kramer post hoc test, multiple comparisons were assessed. Fisher’s exact test was used to examine the significance of the association between the categorical data. All data analyses were performed using the JMP 13 statistical software (SAS Institute, Cary, NC, USA). *P* < 0.05 was considered statistically significant.

### Ethics approval

The study was approved by the Institutional Review Board in Kyushu University in Fukuoka, Japan (Approval number 26-224).

## Supplementary Information

Supplementary Information.

## Data Availability

The datasets generated during and/or analyzed during the current study are available from the corresponding author on reasonable request.
