# Supplementary material for: Nuclear β-catenin translocation plays a key role in osteoblast differentiation of giant cell tumor of bone
Source: Sci Rep. 2022 Aug 4;12:13438. doi: 10.1038/s41598-022-17728-5 (PMC9352730; doi:10.1038/s41598-022-17728-5)
Supplement: Supplementary file 1 — Supplementary Information. [file 41598_2022_17728_MOESM1_ESM.pdf]

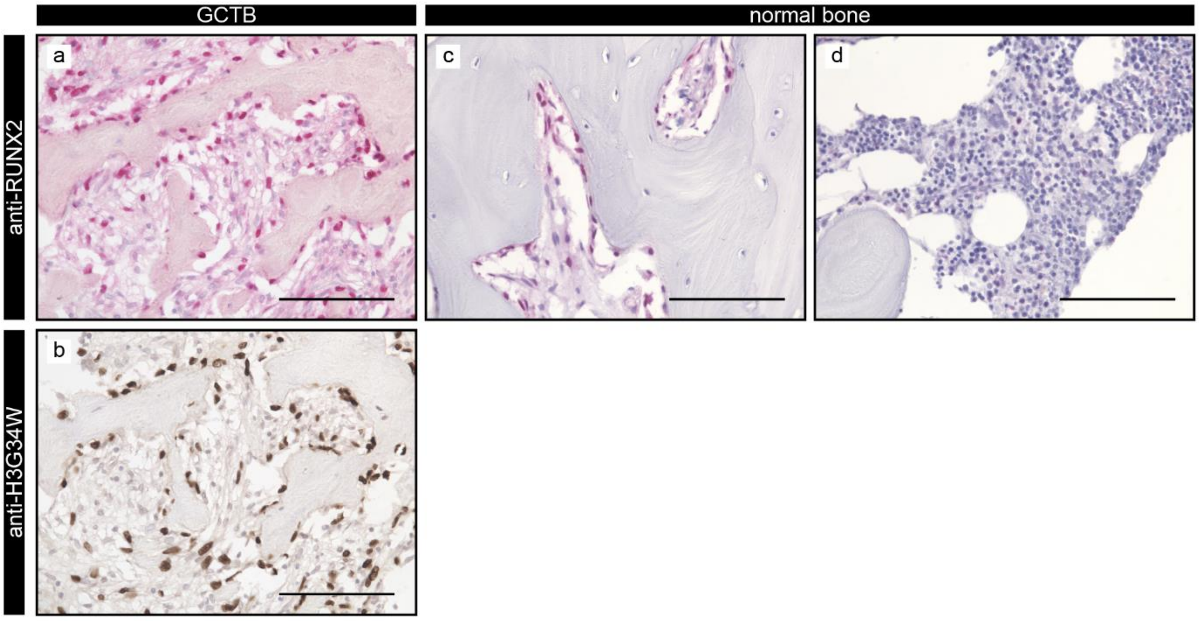


**Supplementary Figure S1: GCTB-SCs express the osteoblast lineage.** (**a, b**) H3G34W-positive and RUNX2-positive cells showed nearly identical distributions in serial sections. (**c, d**) The specificity of the RUNX2 antibody for normal bone tissue. Immunohistochemical staining with RUNX2 was positive for osteoblasts (**c**) while negative for bone marrow cells (**d**). Scale bars, 100 μm.

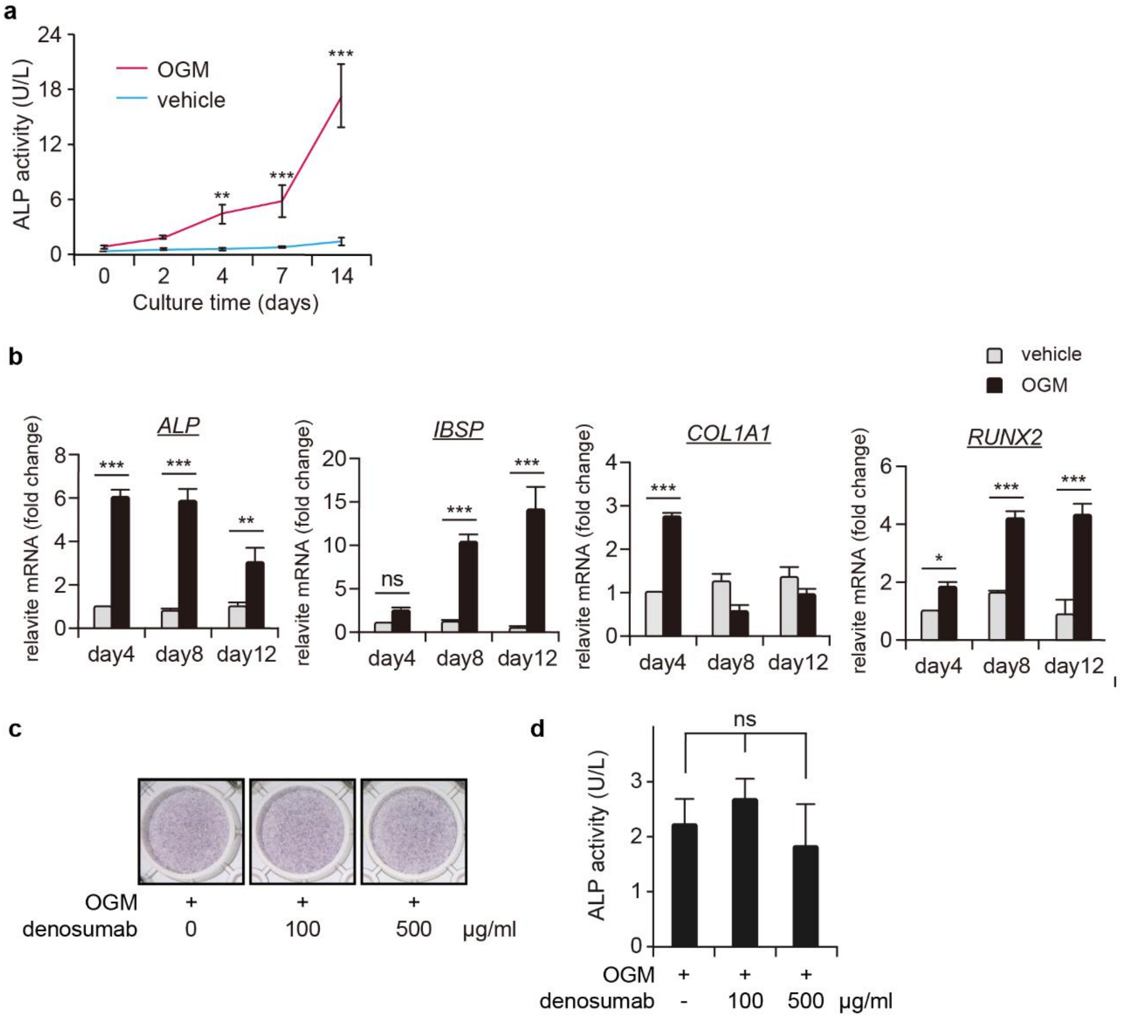


**Supplementary Figure S2: Detailed analysis of osteoblastic characteristics of pGCTB-SCs.** (**a**) OGM stimulated ALP expression of pGCTB-SCs in a time-dependent manner. After pGCTB-SCs were cultured with or without OGM for the indicated periods, the cells were lysed, and intracellular ALP activity was measured. $^{**}P < 0.001$, $^{***}P < 0.0001$ vs vehicle (**b**) Expression of osteoblastic markers was significantly upregulated by induction of differentiation with different timing. The expression of *ALP* and *COL1A1* were evaluated with day4 samples because these markers were upregulated in early-stage of osteoblastic differentiation. Meanwhile, that of *IBSP* and *RUNX2* were assessed with day8 or day12 because these markers increased in the middle to the late stage of the differentiation. Gene expression at each stage is given relative to the level on day 4 without OGM. Values represent means ± SD (n = 4). $^{*}P < 0.05$, $^{**}P < 0.001$, $^{***}P < 0.0001$ vs vehicle. **(c, d)** Denosumab did not affected ALP expression in pGCTB-SCs. Tumor cells were cultured with OGM and 100 or 500 μg/ml denosumab for 1 week, and then cytochemical staining was performed (**c**). Effects of denosumab on ALP expression of pGCTB-SCs were also evaluated by measuring $OD_{405}$ (**d**). Values represent means ± SD (n = 4). ns, not significant.

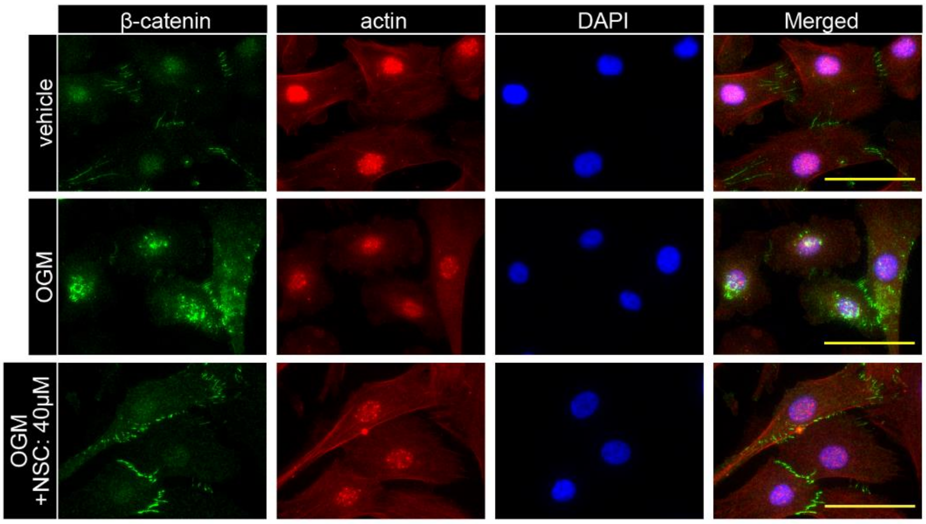


**Supplementary Figure S3: Effect of NSC on pGCTB-SC.** Addition of NSC decreased the OGM-induced nuclear translocation of β-catenin, although the accumulation of β-catenin involved in the cell–cell adhesion was not inhibited. Scale bars, 50 μm.

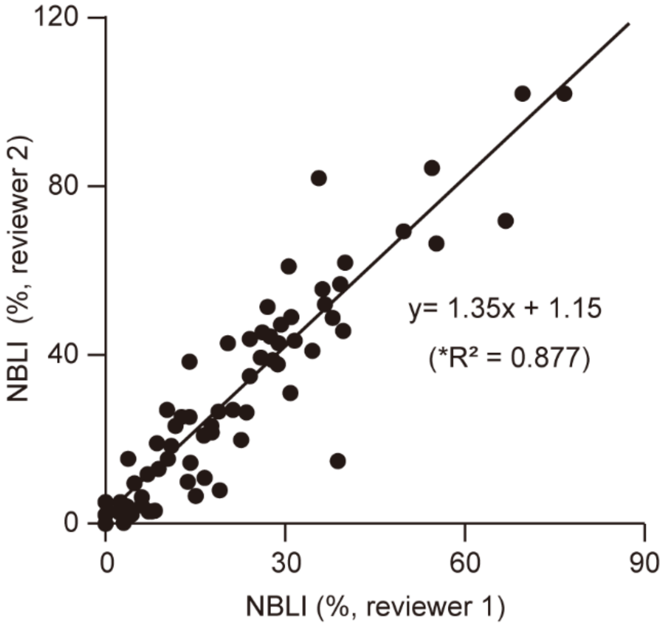


**Supplementary Figure S4: Retrospective evaluation of the distribution of NBLI revealed a significant coincidence among reviewers**. NBLI, nuclear β-catenin labeling index; *P < 0.0001 (Pearson product-moment correlation coefficient).

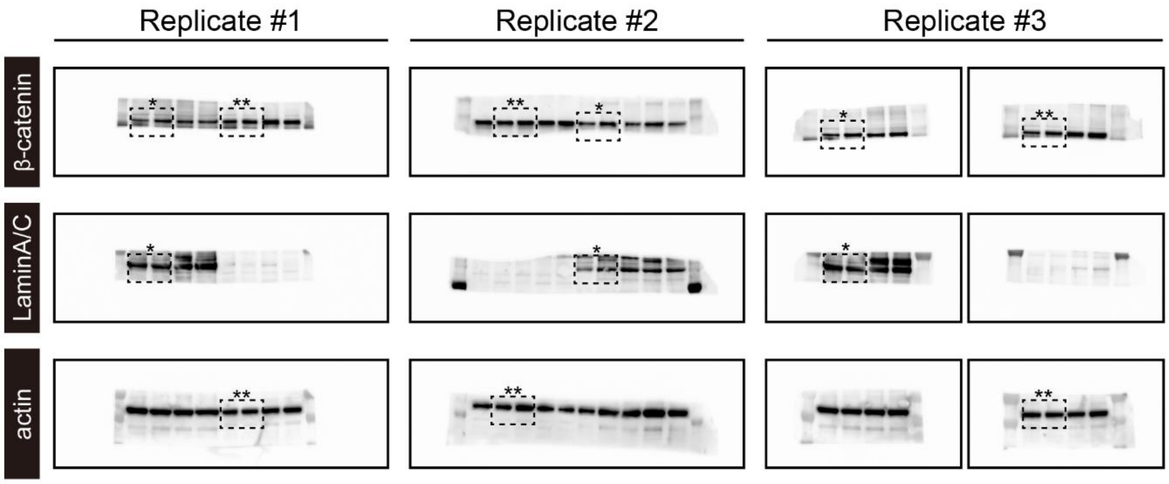


**Supplementary Figure S5: All replicates of Western blotting performed in Figure 2.** The extracted blots in Figure **2a** and **2b** are framed by dot-lines. *, nuclear protein. **, cytoplasmic protein.

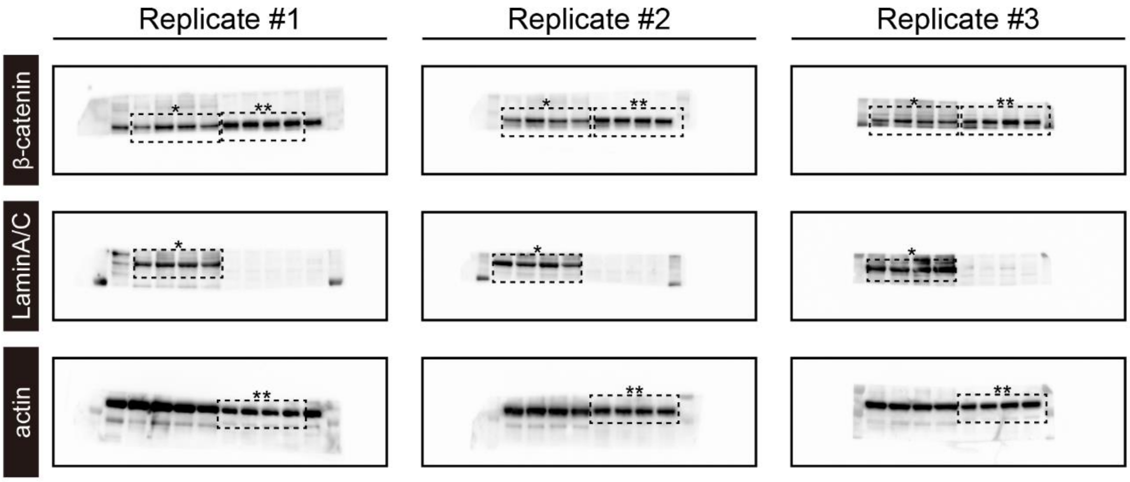


**Supplementary Figure S6: All replicates of Western blotting performed in Figure 3.** The extracted blots in Figure **3d** and **3e** are framed by dot-lines. *, nuclear protein. **, cytoplasmic protein.

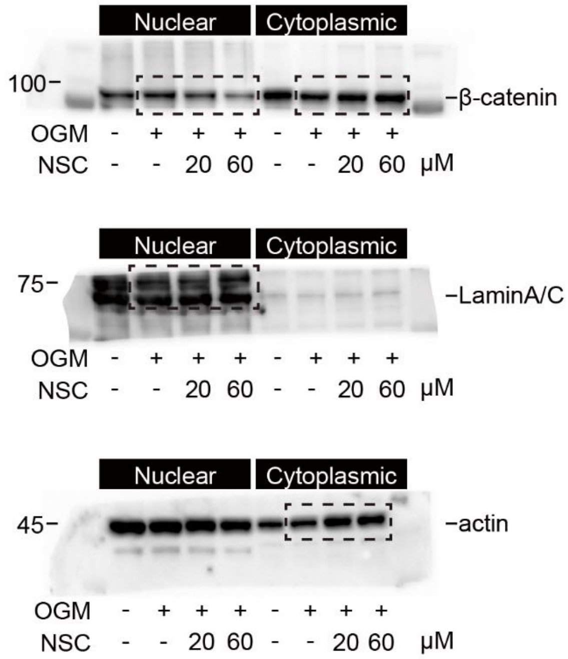


**Supplementary Figure S7: Unedited images of the blots in Figure 4.** The extracted blots of LaminA/C and actin in Figure **4d** are framed by dot-lines.

**Supplementary Table S1.** Background data for 86 GCTB sections

| | |
|---|---|
| *Patient age (years) | 37.1 ± 15.8 |
| Sex (female, %) | 48 (55.8) |
| Affected site (n) | Femur (27) |
| | Tibia (24) |
| | Radius (8) |
| | Fibula (7) |
| | Humerus (6) |
| | Spine (7) |
| | Others (7) |

*Data are presented as means ± SD.

**Supplementary Table S2.** Human-specific primer pairs used qRT-PCR

| Gene | Forward Primers | Reverse Primers |
|---|---|---|
| *ALP* | 5′-TTGGGGTGCACCATGATTTC-3′ | 5′-TTCAGTGTCTCTTGCGCTTG-3′ |
| *BGLAP* | 5′-ACCGAGACACCATGAGAGCC-3′ | 5′-CTGCTTGGACACAAAGGCTCG-3′ |
| *COL1A1* | 5′-TTGACCAACCGAACATGACC-3′ | 5′-TTCAAGCAAGTGGACCAAGC-3′ |
| *GAPDH* | 5′-AATTCCATGGCACCGTCAAG-3′ | 5′-ATCGCCCCACTTGATTTTGG-3′ |
| *IBSP* | 5′-ATTTTGGGAATGGCCTGTGC-3′ | 5′-TCGTGGCCTGTACTTAAAGACC-3′ |
| *LEF1* | 5’-AGGAATCTGCATCAGGTACAGG-3’ | 5’-TTTGCACGTTGGGAATGAGC-3’ |
| *RUNX2* | 5′-TCAGCTAAAAGCCGCACATG-3′ | 5′-TGTTTGGTAAGGCTGGTTGG-3′ |
